# Transmitted drug resistance, selection of resistance mutations and moderate antiretroviral efficacy in HIV-2: Analysis of the HIV-2 Belgium and Luxembourg database

**DOI:** 10.1186/1471-2334-8-21

**Published:** 2008-02-27

**Authors:** Jean Ruelle, François Roman, Anne-Thérèse Vandenbroucke, Christine Lambert, Katrien Fransen, Fedoua Echahidi, Denis Piérard, Chris Verhofstede, Kristel Van Laethem, Marie-Luce Delforge, Dolorès Vaira, Jean-Claude Schmit, Patrick Goubau

**Affiliations:** 1 Université Catholique de Louvain, AIDS Reference Laboratory, Avenue Hippocrate 54 -5492, 1200 Bruxelles, Belgium; 2 CRP Santé, Laboratoire de Rétrovirologie, Val Fleuri 84, 1526 Luxembourg, Luxembourg; 3 Institute of Tropical Medicine, AIDS Reference Laboratory, Nationalestraat 155, 2000 Antwerpen, Belgium; 4 Universitair Ziekenhuis Brussel, AIDS Reference Laboratory, Laarbeeklaan 101, 1090 Bruxelles, Belgium; 5 UGent, AIDS Reference Laboratory, DePintelaan 185 Blok A, 9000 Gent, Belgium; 6 University Hospitals Leuven, AIDS Reference Laboratory, Herestraat 49, 3000 Leuven, Belgium; 7 Hôpital Erasme, AIDS Reference Laboratory, Route de Lennik 808, 1070 Bruxelles, Belgium; 8 CHU Liège, AIDS Reference Laboratory, Domaine Universitaire du Sart-Tilman B35, 4000 Liège, Belgium

## Abstract

**Background:**

Guidelines established for the treatment of HIV-1 infection and genotype interpretation do not apply for HIV-2. Data about antiretroviral (ARV) drug efficacy and resistance mutations is scarce.

**Methods:**

Clinical data about HIV-2 infected patients in Belgium and Luxembourg were collected and the effect of ARV therapy on plasma viral load and CD4 counts were analysed. Viral RNA encoding for protease (PR) and reverse transcriptase (RT) from ARV-naïve and treated patients were sequenced.

**Results:**

Sixty-five HIV-2 infected patients were included in this cohort. Twenty patients were treated with 25 different ARV combinations in a total of 34 regimens and six months after the start of ARV therapy, only one third achieved viral load suppression. All of these successful regimens bar one contained protease inhibitors (PIs). Mean CD4 gains in the group of viral load suppressors and the group of patients treated with PI-containing regimens were respectively significantly higher than in the group of non-suppressors and the group of PI-sparing regimens. The most frequent mutations selected under therapy (compared to HIV-2 ROD) were V71I, L90M and I89V within PR. Within RT, they were M184V, Q151M, V111I and K65R. All of these mutations, except K65R and M184V, were also found in variable proportions in ARV-naïve patients.

**Conclusion:**

Despite a high rate of ARV treatment failure, better virological and immunological results were achieved with PI-containing regimens. The analysis of polymorphic positions and HIV-2 specific mutations selected during therapy showed for the first time that transmission of drug resistant viruses has occurred in Belgium and Luxembourg. The high heterogeneity in ARV combinations reflects a lack of guidelines for the treatment of HIV-2 infection.

## Background

Human immunodeficiency virus type 2 (HIV-2) is a lentivirus that causes AIDS [1]. Compared to HIV-1, the disease progression is slower [2] and the transmission rate and plasma viral load are also lower [3,4]. Most of the patients infected with HIV-2 are asymptomatic and do not need antiretroviral (ARV) therapy if HIV-1 guidelines are used as a reference [5]. If ARV therapy is started, the choice of drug regimens is limited due to lower drug susceptibilities in comparison to HIV-1. Firstly, HIV-2 is naturally resistant to the non-nucleosidic reverse transcriptase inhibitors (NNRTI) [6,7] and to the fusion inhibitors (FI) that are available on the market [7]. Secondly, reduced susceptibility to some protease inhibitors (PI) has previously been described. HIV-2 displays resistance to amprenavir (APV) [7-9]. Reduced efficacy of nelfinavir (NFV) has been observed in primary isolates from patients [10] and *in vivo *[11]. In vitro, IC_50 _to atazanavir (ATV) and tipranavir (TPV) are higher compared to HIV-1, while IC_50 _to lopinavir (LPV) and darunavir (DRV) are within the same range [12].

The majority of HIV-2 infected persons live in West African countries [13], where HAART is not yet available or has only been implemented recently. No large scale clinical studies have been published on the immunological and virological effects of ARV drugs. Furthermore, there is no consensus for plasma viral load quantification and no commercial assay is available. As a consequence, the interpretation of ARV impact on viral replication raises problems. A first evaluation of viral load measurement techniques [14] as well as the use of an HIV-1 designed kit for HIV-2 RNA quantification has been documented [15]. Some clinical studies based on variable cohort sizes and designs have been made. ARV therapy has shown to have a modest impact on CD4 cell recovery [11,16-18]. Better outcomes were seen with PI-containing regimens in some studies [16,19,20], but others found no difference [17,21].

Although viral evolution occurs slowly in HIV-2 infection [22], the appearance of mutations in the protease (PR) and the reverse transcriptase (RT) genes is common under drug pressure. In HIV-2, these two drug targets harbour amino acid residues which are also involved in HIV-1 drug resistance: 10V, 32I, 36I, 46I, 71V in the protease gene [23] and 118I, 215S in the RT gene in addition to the 3 positions linked to the NNRTI resistance (181I, 188L and 190A) [6,24]. Some mutations appearing under treatment have been clearly linked with therapeutic failure. In the protease, changes were observed that have already been described for HIV-1 drug resistance (10I, 47A, 50V, 54M, 71I, 82F/L, 84V, 90M) in addition to HIV-2 specific positions or substitutions (33L, 45R, 56A, 62A, 99F) [10,25-28]. The number of mutations needed to confer high-level resistance to PIs is lower in HIV-2 [9]. In the RT, the K65R mutation was selected under tenofovir containing regimens [29] or in combination with Q151M and M184V under stavudine, abacavir or didanosine therapy. The Q151M mutation, which is frequently found [30], has been documented under different NRTI-containing regimens while M184V was linked to lamivudine (3TC) use [16,24,28,31,32]. Transmission of drug resistant strains may have occurred [33] as well as viruses with a mutational pattern facilitating the acquisition of multi-drug resistance [9,10]. In this observational study, a small cohort of HIV-2 infected patients is presented. A positive effect of ARV therapy on clinical parameters was observed, but the impact on CD4 recovery was modest and virological failures were frequent. Drug resistance mutations were detected at failure and some of these mutations were already present in ARV-naïve patients.

## Methods

### Data Collection

An anonymous data sheet was sent to the clinicians in charge of HIV-2 infected patients using the Belgian AIDS Reference Laboratories network and the Laboratoire de Rétrovirologie in Luxembourg. Patients were identified by a code. Patients with an untypeable HIV status in immunoblot or presumably co-infected with HIV-1 and HIV-2 were excluded. In a first round, epidemiological and clinical data were collected as well as the availability of retrospective samples. In a second round, frozen plasma or serum samples stored in the normal diagnosis and follow-up settings, were obtained for the sequencing of PR and RT coding regions at different points during the treatment.

### Viral load measurements

Two different methods were used for plasma viral load assessment.

On the one hand, HIV-2 plasma viral load was measured by a real-time PCR in-house assay on a Lightcycler platform [34]. The assay was modified to obtain a sensitivity of 50 RNA copies per millilitre and it used a quantified synthetic RNA as external standard. Viral RNA was extracted from 1 millilitre of plasma or serum by Nuclisens Magnetic Isolation kit on a Mini-Mag apparatus (Biomérieux, Boxtel, The Netherlands). RNA was eluted in 40 μl buffer. 8 μl were used for reverse transcription using the Transcriptor first strand cDNA synthesis kit (Roche Diagnostics, Penzberg, Germany) with random hexamers (final concentration 60 μM) in a final volume of 20 μl. The real-time PCR was performed using the Lightcycler FastStart DNA Master^PLUS ^SYBR Green I, 100 μl kit (Roche Diagnostics, Penzberg, Germany) on a Lightcycler 2.0 platform. Each capillary contained 48 μl PCR grade water, 6 μl of each primer (in a final concentration of 0.6 μM – see ref 30 for sequences), 20 μl of Master Mix provided in the kit and 20 μl cDNA. Each run consisted of forty cycles of amplification (95°C 15 s – 60°C 40 s – 72°C 30 s) followed by a melting curve analysis.

On the other hand, a semi-quantitative assay was used as described by Van Kerckhoven *et al*. [35] with HIV-2 specific primers described in [36]. The latter method was the reference in Belgium before 2004 and is still used for the follow-up of some patients.

### Genotype

RT-PCR and inner PCR were performed as described by Colson *et al*. [27] and PCR products were analysed in a 2% agarose gel with ethidium bromide. This protocol generated a 1507 bp fragment which covers the protease and the RT coding regions. Viruses of subtype B failed to be amplified. In this case, two separate inner PCR reactions were performed using 2 μl of RT-PCR product. One reaction amplified the RT coding region with the forward primer JR23 5'-TAATGACAGGCGACACC-3' and the reverse primer JR24 5'-TGTGCTGCCCAATTTAG-3', both at a final concentration of 0.2 μM. PCR conditions were as follows: 10 min at 94°C, followed by 40 cycles of 30 s at 94°C, 45 s at 57°C, 1.5 min at 72°C, and finally an incubation of 10 min at 72°C. The second reaction amplified the protease coding region, with the forward primer JR21 5'-AGACACCATACAGGGAGC-3' and the reverse primer JR41 5'-TGTATGGATTAGTAGGAGGCG-3' under the same experimental conditions.

PCR products were then purified using the QIAquick PCR purification kit (QIAGEN, Hilden, Germany). Twenty nanograms of each PCR product were sequenced with the BigDye Terminator v1.1 cycle sequencing kit (Applied Biosystems, Foster City, CA, USA). Primers used for sequencing of PCR products obtained by the first protocol were H2Mp3 [27] and JR 40 5'-AGGATTAGTTGGAGGTGC-3' for the protease coding region, and JR23, JR24, JR25 5'-GCACCTCCAACTAATCCT-3' and JR 26 5'-GCAGTATATGGTCTAAAGTC-3' for the RT coding region. If the first PCR protocol was not successful, the protease coding region was sequenced with primers JR21 and JR41, while the RT coding region was sequenced with JR23, JR34 5'-AGTTGAGCTGCCCAATTTAA-3' and JR35 5'-CGCCTCCTACTAATCCATACA-3'. Products of the sequencing reaction were purified by ethanol-acetate precipitation and analysed on an ABI Prism 310 genetic analyser (Applied Biosystems).

### Statistical analysis

Statistical analysis was performed using the JMP software version 6 (SAS institute, Cary, NC, USA). The significant effect of ARV therapy on plasma viral load was assessed using a Kaplan-Meyer survival curve in order to consider if virological failure was the outcome at three different time reference points after therapy initiation. To interpret the impact of ARV therapy on CD4 count evolution, data was first standardised to obtain CD4 values at the same reference points for every therapy, using a linear regression model to interpolate values between existing measurements. In a first model, delta CD4 values were calculated from treatment initiation at intervals of three months. Two different parameters were tested: the therapy (PI-containing or not) and virological failure. Analysis of the variance between groups was done using a Tukey test. In a second model, the raw CD4 data on a time scale between the two different therapy groups was considered and response specification was assessed by constructing linear combinations with a Manova fit model.

### GenBank accession numbers

Earliest samples from 30 patients were submitted to GenBank and received accession numbers from EF611309 to EF611338: the first 23 were ARV-naïve (from EF611309 to EF611331) and the last 7 were treated patients.

### Ethical approval

This evaluation was approved by the ethical committee of the Medical Faculty of the Université Catholique de Louvain.

## Results

### Epidemiological data

Sixty-five patients were included in our cohort and data is summarised in Table 1. The male/female ratio was about 1, median age was 42 at the end of 2006, and the transmission route was essentially heterosexual. The majority (i.e. 63%) of cases were of West African origin, although cases from Europe, central Africa and Asia were recorded. Twenty out of the 65 patients received ARV therapy and 15 were still on therapy at the end of 2006.

**Table 1:** Epidemiological data about the 65 patients from Belgium and Luxembourg.

Gender	Female	31 (48.5%)
	Male	33 (51.5%)
	Unknown	1
Age	Mean	42.84 years (range from 25 to 71)
	Median	42 years

Countries of origin	West Africa	29 (63%)
	Ghana	8
	Ivory Coast	7
	Cape Verde Islands	5
	Guinea-Bissau	3
	Guinea	2
	Senegal	1
	Liberia	1
	Nigeria	1
	Burkina-Faso	1
	Europe	11 (24%)
	Belgium	6
	Portugal	5
	Central and Southern Africa	4 (8.5%)
	Democratic Republic Congo	3
	South-Africa	1
	Asia: Nepal	2 (4%)
	Not documented	19

Transmission	Heterosexual	37 (86%)
	Homosexual (MSM)	2 (4.5%)
	Intra-venous drug use	2 (4.5%)
	Transfusion	2 (4.5%)
	Not documented	22

Year of infection	Between 2002 and 2006	10 (33%)
	Between 1997 and 2001	7 (23%)
	Before 1997	13 (43%)
	Not documented	22

ARV use	Treated	20 (31%)
	Exposed to NRTIs only	5
	Exposed to NRTIs and PIs	15
	Treated at the end of 2006	15
	Never been treated	45 (69%)
Subtype	A	25 (83%)
	B	5 (17%)
	Not documented	35

The percentages were calculated for patients with available data (collected until 2006-12-31). ARV: antiretroviral therapy

### Effect of ARV therapy on plasma viral load

As different methods were used for the viral load determination, and in the absence of a gold standard, we chose qualitative criteria to evaluate the efficacy of therapy. Either the regimen successfully suppressed viral replication and plasma viral load became undetectable, or replication was not suppressed and this was defined as virological failure. The threshold used was the detection limit of the least sensitive method. All therapies were started in the presence of a detectable viral load and baseline viral load values are given in Table 2. The presence or absence of viral replication was investigated at three points: 6, 12 and 24 months after initiation of therapy. Overall, 60%, 53% and 50% of virological failures were observed respectively. Details are available in Table 2. Twenty out of 34 regimens were first line therapies, and 7 second-line therapies were initiated in the presence of resistance mutations. A Kaplan-Meier survival curve was drawn to compare the appearance of virological failure in PI-containing and PI-sparing regimens (not shown). A significant difference existed between the two (p = 0.02): the mean times to virological failure were 9.42 months for the PI-containing group and 1.2 months for the PI-sparing group.

**Table 2:** Effect of ARV therapy on plasma viral load levels after 6, 12 and 24 months.

		**Baseline VL**	**VL 6 m**	**VL 12 m**	**VL 24 m**	**Baseline CD4**	**Delta CD4 12 m**
**PI-containing regimens**							

1	AZT-3TC-LPV/r	3.65	U	U	U	40	+ 323
2	d4T-3TC-IDV/r	2.36	U	U	U	490	+ 231
3	d4T-ABC-LPV/r	5.36	**D**	**D**	**D**	338	+180
4	3TC-TDF-SQV-ATV/r	5.73	U	U	NA	92	+161
5	d4T-3TC-NFV	3.36	U	U	U	261	+147
6	TDF-FTC-ATV/r	5.40	D	NA	NA	10	+140
7	AZT-3TC-LPV/r	4.05	U	U	U	62	+136
8	d4T-3TC-IDV	NA	NA	NA	NA	527	+ 89
9	d4T-3TC-LPV/r	4.36	U	U	U	128	+ 67
10	ddI-TDF-IDV/r	3.60	D	U	NA	120	+ 50
11	d4T-3TC-NFV	4.36	**D**	**D**	**D**	380	+ 49
12	AZT-3TC-FPV/r	3.49	**D**	D	NA	290	+ 40
13	AZT-3TC-IDV	5.29	U	U	D	280	+ 21
14	d4T-3TC-NFV	4.36	D	**D**	NA	257	- 14
15	3TC-TDF-IDV/r	2.40	**D**	**D**	NA	180	- 31
	Mean	4.13				230	+106
	Median	4.21				257	+89

**PI-sparing regimens**							

16	AZT-3TC-ABC	3.01	**D**	**D**	**D**	150	+ 57
17	d4T-3TC-ABC-TDF	3.36	U	U	U	630	+12
18	ddI-TDF-ABC	4.05	**D**	**D**	**D**	174	- 8
19	AZT-3TC-ABC	5.07	**D**	**D**	NA	166	- 53
20	AZT-3TC-ABC	2.40	D	NA	NA	737	- 60
21	3TC-ABC-TDF	3.36	NA	D	NA	195	- 61
22	AZT-3TC-ABC	4,78	**D**	**D**	**D**	280	- 62
	Mean	3.72				333	-25
	Median	3.36				195	.53

**Regimens not included in the comparison above**							

23	AZT-3TC-NVP	NNRTI is not active, suboptimal bitherapy					
24	AZT	Suboptimal monotherapy					
25	ddI-d4T-EFV	NNRTI is not active, suboptimal bitherapy					
26	AZT-3TC-NFV	Therapy switched after 1 month					
27	3TC-ABC-FPV/r	Therapy switched after 1 month					
28	AZT-3TC-SQV/r	Therapy started end of 2006					
29	AZT-3TC-LPV/r	Therapy started end of 2006					
30	AZT	Pregnancy, prevention MTCT					
31	AZT	Pregnancy, prevention MTCT					
32	AZT-3TC-LPV/r	Pregnancy, prevention MTCT					
33	3TC-d4T	Lost to follow-up					
34	ddI-d4T-ABC-SQV-NFV	Lost to follow-up					

Regimens were classified within the PI-containing and PI-sparing groups from the highest to the lowest delta CD4 counts after 12 months of therapy.

Baseline viral load levels are expressed in log copies/ml of plasma, CD4 counts are expressed in cells/mm^3^.

All regimens are first line therapies, except regimens 3, 4, 7 and 18 to 22 which are second line therapies; 6, 10, 17, 21 are third line therapies and 15 is a fourth line therapy.

U = undetectable plasma viral load; D = detectable viral load; NA = not available (unavailable sample or end of the treatment); MTCT = mother to child transmission.

### Effect of ARV therapy on CD4 count

Information on CD4 counts evolution was gathered for 25 therapies. Three suboptimal therapies were excluded from the statistical analysis (Table 2, lines 23–25). Mean CD4 at baseline was 263 cells/mm^3 ^(ranging from 10 to 737). In the group of patients who had never been treated, data from 10 patients with baseline CD4 counts between the same ranges were considered, with a mean at the first visit of 402 cells/mm^3 ^(193–660). Over one year, the mean loss was 16 CD4/mm^3^.

The CD4 gain after therapy initiation was plotted on a time scale. The PI-containing and PI-sparing groups, as well as the viral load suppressor and non-suppressor groups, were compared (Table 3). A Tukey test was performed, comparing the variance in CD4 gain at intervals of 3 months after therapy initiation. Viral load suppressors had a distinctively higher CD4 gain than viral load non-suppressors (p < 0.0001), with respective mean gains of 137 and 23 CD4/mm^3 ^after 12 months. Differences in treatment had a significant influence on CD4 gain (p = 0.003). A mean gain of 106 CD4/mm^3^/year was observed in the PI-group while a mean loss of 25 CD4/mm^3^/year was obtained in the PI-sparing group. A second test was performed to establish the immunological response based on raw CD4 data using a Manova test. After 12 months of treatment, a gain of 96 CD4/mm^3 ^in the PI-group and a loss of 11 CD4/mm^3 ^in the PI-sparing group had been recorded. For the PI-group, this gain was already observed after 3 months of therapy and was maintained afterwards while the decrease observed for the PI-sparing group did not significant differ from zero.

**Table 3:** Effect of ARV therapy on CD4 counts after 12 months.

	**BASELINE CD4 (cells/mm^3^)**				**DELTA CD4 after 12 months**				
		
	**Mean**	**Median**	Interval		**Mean**	**Median**	Interval		
Non-treated (N = 10)	**402**	**427**	193	660	**-16**	**-21**	-112	64	
ARV therapies (n = 22)	**263**	**226**	10	737	**64**	**50**	-62	323	
- with PIs (n = 15)	**230**	**237**	10	527	**106**	**89**	-31	323	p = 0,0003
- without PIs (n = 7)	**333**	**195**	150	737	**-25**	**-53**	-62	57	
- undetectable VL (n = 8)	**248**	**195**	40	630	**137**	**141**	12	323	p < 0,0001
- detectable VL (n = 14)	**272**	**226**	10	737	**23**	**16**	-62	180	

Interval columns give minimum and maximum CD4 cells/mm^3^. N is the number of patients for the untreated group, and n is the number of therapies in the treated group. p values were obtained by the analysis of variances using a Tukey test (see Methods section). PI = protease inhibitor, VL = plasma viral load.

Finally, baseline CD4 counts were stratified in three groups, more than 400, between 400 and 200 and less than 200 cells/mm^3^. The same tendencies were observed for respectively 4, 7 and 11 different treatments but they had poor statistical relevance, especially for the groups higher than 200 CD4/mm^3^.

### Mutations selected on therapy, polymorphisms and resistance at baseline

Table 4 shows the mutations that appeared in the PR and RT during ARV therapy. The ROD strain (subtype A) was used as reference for the description of mutations.

**Table 4:** Mutations selected on ARV therapy.

**Patient**	**ARV drug regimens**	**Mutations selected in the PR**	**Mutations selected in the RT**	**Corresponding line in table 2**
A	d4T-3TC-NFV	V62A, V71I, L99F	M184V	14
	d4T-ABC-LPV/r	+ S43I, K45R, V47A, I89V	+ I10V, K35T, K82R, Y115F, Q151M	3
B	AZT-3TC-ABC		M184V	20
	d4T-3TC-ABC-TDF		+ A62V, K65R, V111I	17
C	AZT-3TC-NVP		Q151M, M184V	23
	ddI-TDF-ABC		+ K65R, N69T, V111I, D218E	18
	3TC-ABC-TDF		+ I90V, S215T	21
D	d4T-3TC-NFV	T56A, V62M, V71I	M184V	11
E	d4T-3TC-NFV	V71I/T (*)		5
F	3TC-ABC-FPV/r	I54M, I89V, L90M	M184V	27
G	AZT-3TC-ABC		K65R, N69S, V83I, I90V, V111I, M184I/V, F214L, Q151M, Y115F	16
H	AZT-3TC-ABC		M184V	22
	TDF-FTC-ATV/r	L90M		6
I	AZT-3TC-ABC		M184V, K70N, K35R, K64R, K82R, Q151M	19

In the PR, the most frequent mutation was V71I: it showed 3 times under NFV therapy. This mutation was associated once with V62A and L99F, once with T56A and V62M and in both cases with a plasma viral load higher than 3 log copies per ml. In the third case V71I was detected as part of a combination with V71T before viral load suppression. The I89V and L90M mutations were each detected twice, the latter under an ATV/r containing regimen and in a FPV/r containing regimen in association with I54M and I89V mutations.

In the RT, the most frequent mutation selected on therapy was M184V. It appeared in eight different treatment failures with different regimens all containing 3TC, which was also the most prescribed ARV drug.

The Q151M mutation was found in viruses from 4 patients with different regimens. Firstly, in association with I10V, K35T, K82R, Y115F and M184V in patient A who had a history of AZT, 3TC, d4T and ABC exposure. Secondly, Q151M appeared in association with the M184V mutation under AZT/3TC pressure (patient C). Thirdly, in patient G, Q151M was observed in association with M184V and F214L under AZT/3TC/ABC pressure. The further continuation of this regimen subsequently resulted in the appearance of K65R, N69S, V83I, I90V and V111I. Lastly, and again under AZT/3TC/ABC pressure (patient I), the Q151M appeared on a M184V background after the disappearance of K70N and with the concomitant presence of K35R, K64R and K82R.

The K65R mutation was selected 3 times on therapy and the only drug in common for the 3 regimens was ABC.

**Figure 1:**
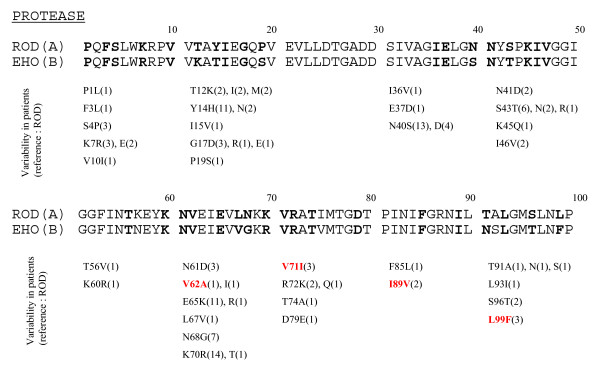
**Variations found in the HIV-2 protease**. Polymorphisms of the PR sequenced from 20 antiretroviral-naïve patients are compared to ROD and EHO strains (respectively subtypes A and B). **X**(bold): amino acid residue where variability was found in ARV-naïve patients. (N): number of samples from naïve patients harbouring the mutation. Red: mutations that were selected under ARV therapy in this study (Table 4).

**Figure 2:**
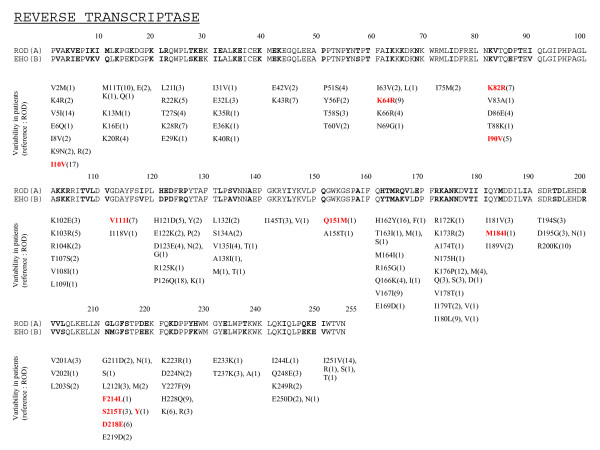
**Variations found in the HIV-2 reverse transcriptase**. Polymorphisms of RT from 23 antiretroviral-naïve patients are compared to ROD and EHO strains (respectively subtypes A and B). **X**(bold): amino acid residue where variability was found in ARV-naïve patients. (N): number of samples from naïve patients harbouring the mutation. Red: mutations that were selected under ARV therapy in this study (Table 4).

Fig. 1 and 2 show the variations found in the PR and the RT, respectively from 20 and 23 ARV-naïve patients. In the PR, the most polymorphic positions were 14, 40, 65, 68 and 70. The mutations V62A, V71I, I89V and L99F, all linked to virological failure, were found in viruses from naïve patients.

In the RT, the most polymorphic positions were 5, 10, 11, 28, 64, 82, 111, 121, 123, 126, 162, 167, 176, 180, 200, 227, 228 and 251, with 7 or more variants out of 23 sequences from different naïve patients. Note that the Q151M mutation was also found in a virus from a recently diagnosed naïve patient originating from Belgium. Phylogenetic analysis of the RT sequences (not shown) showed that this virus was related to another one also harbouring the Q151M mutation. The patients affected were the only 2 homosexual men of this group. Furthermore, positions 10, 35, 64, 69, 82, 83, 90 and 111 were found in variable proportions in naïve patients (see Fig 2 for details). The M184I mutation was found in one ARV-naïve patient but was present in the provirus only.

## Discussion

We describe retrospective data on 65 HIV-2 infected patients, with insights on the effect of ARV therapy on plasma viral load, CD4 counts and the selection of drug related mutations.

In regard to the epidemiological picture of this cohort, the main characteristics do fit with the trends described in literature: the majority of patients originate from West Africa, the transmission is essentially heterosexual, and the male/female ratio is about 1 [3,37]. We also recorded patients originating from countries where the presence of HIV-2 had not yet been reported, such as R.D. Congo, South-Africa and Nepal. Although Central and Southern Africa is confronted with a huge HIV-1 epidemic, HIV-2 is not completely absent [38].

The impact of ARV therapy on viral replication was studied using a qualitative marker: the presence or absence of a detectable plasma viral load. Up until now several assays had been described for HIV-2 quantification [15,34,39-42], the majority of them using real-time PCR. But even among these techniques, the standards used differ, resulting in important discrepancies in absolute quantification [14].

We observed that viral load suppression was achieved with PI-containing regimens in 50% of the therapies after six months of treatment. Only one therapy with 4 NRTI resulted in an undetectable viral load within the same period. This study faced three main drawbacks. Firstly, the number of patients recorded was limited and many different ARV combinations were given. We do know that some drugs have a reduced activity against HIV-2 and their impact in the PI- containing or sparing regimens could have influenced the results. Secondly, we had no data on adherence but there were no indications for a different adherence in the PI and non-PI subgroups. Thirdly, long term efficacy of ARV drugs on viral load suppression was difficult to evaluate as a failing regimen was stopped as soon as the plasma viral load results became available to the clinician. Nevertheless, long term viral suppression was observed only within PI-containing regimens. The viral load levels at baseline could not explain this difference as they were higher in the PI-treated group. This observation is consistent both with the knowledge we have of HAART in HIV-1, where guidelines recommend the administration of 2 different classes of drug [5], and with the CD4 count evolution in our group of patients.

The CD4 gain was significantly greater if PIs were included in the therapy compared to regimens with RTIs only. We observed a mean loss of 16 CD4/mm^3 ^per year in a group of 10 non-treated patients. This loss is similar to what was found elsewhere for HIV-2 [43]. The CD4 loss in untreated patients did not differ significantly from the loss observed in the subgroup of virological failures or from the subgroup treated without PIs. Our study further indicates that the best results in terms of immunological reconstitution were obtained when the plasma viral load was fully suppressed. Other studies have found a CD4 gain in HIV-2 treated patients between 41 [17] and 163 [11] cells per year. If we consider all the patients treated with 3 or more ARV studied here, the CD4 gain of 64 CD4/mm^3 ^per year seems modest. Several hypotheses may explain this moderate efficacy. First, PR and RT catalytic sites differ between HIV-1 and HIV-2 despite a common structural backbone at protein level and therefore, the sensitivity of HIV-2 to drugs differs from HIV-1 [6,7,44]. Some of the regimens observed include NFV and APV, to which sensitivity is reduced [7,8,11].

Moreover, we know that the plasma viral load is lower in HIV-2 infection compared to HIV-1 in spite of an equivalent proviral load [45]. The viral replication cycle may differ from HIV-1, with a lower number of infective cycles. Drugs now available target the replicative cycle and may be therefore less active. Finally, there are no criteria or guidelines to start therapy based on biological parameters and more clinical studies are needed to determine the optimal conditions for a successful HIV-2 therapy.

The use of suboptimal regimens will prompt the appearance of resistant viruses. Here, we observed mutations in the reverse transcriptase that had already been described before: K65R, Q151M and M184V [28-32]. These were the mutations most frequently observed under therapy. We also noted the frequent occurrence of the V111I mutation: this mutation was present in 7 out of 23 ARV-naïve patients, but also showed 3 times in association with K65R under various NRTI-only regimens. A major challenge with genotype interpretation is the discrimination between natural polymorphisms and changes affecting sensitivity to ARV drugs. Changes that appeared under therapy at RT positions 10, 64, 82, 90, 111, 167 and 218 (see Fig. 2) were widely found in ARV-naïve patients [46]. Phenotypic studies are needed to assess their impact on resistance. For instance, the K35R mutation, already described elsewhere [24], was selected under AZT/ABC/3TC therapy but was also present in a naïve patient. The K70N, also selected on AZT/ABC/3TC therapy was observed *in vitro *when ROD and EHO HIV-2 strains were cultured under ABC pressure [47]. We observed no selection of Y at position 215 [48]. S215T was selected in 3TC/ABC/TDF therapy, but 215T and 215Y were also observed in naïve patients.

In the PR, the appearance of mutations was associated with the 3 regimens that included NFV. The V71I mutation was common to the 3 cases. This same mutation was also retrieved in 3 others naïve patients. The V47A mutation was observed elsewhere under LPV/r treatment [49]. We confirmed the presence of this mutation by LPV/r in one case of second line virological failure.

It is known that the L90M mutation occurs *in vitro *under SQV [25,47] and NFV pressure [9], and *in vivo *during NFV, IDV, SQV/r and LPV/r treatments [26,49]. We found presence of L90M under FPV/r and ATV/r containing regimens, suggesting that L90M is a multi-resistance mutation.

Although more phenotypic and clinical studies are needed to assess the impact of mutations in more detail, our observations suggest a low genetic barrier to resistance. The use of a PI-based regimen, which our results tend to recommend, could be limited by the selection of multi-resistant strains.

Besides substitutions that are not clearly linked to resistance such as K35R, I90V and V111I in the RT, we observed viruses or proviruses from naïve patients harbouring known resistance mutations like Q151M and M184I. In the protease, we found mutations at positions 62, 71, 89 and 99 in viruses from ARV naïve patients, positions which are possibly linked to resistance [9,27]. This indicates for the first time that transmission of resistant strains does occur in Belgium and Luxembourg.

## Conclusion

This observational study showed that treatment of HIV-2 infection is heterogeneous in Belgium and Luxembourg and has moderate efficacy. Immune recovery was observed with PI-containing drug combinations when plasma viral load was suppressed. Selection of resistance mutations occurred in both PR and RT. Analysis of polymorphisms in ARV naïve patients and mutations appearing during therapy showed for the first time that transmission of resistant HIV-2 strains has occurred in Belgium and Luxembourg. This implies a reduced usefulness of ARV for the treatment of HIV-2 infections in the future. There is therefore an urgent need for genotype interpretation rules as well as standardisation and implementation of HIV-2 laboratory techniques.

## Competing interests

The author(s) declare that they have no competing interests.

## Authors' contributions

JR coordinated the data and samples collections from the different Belgian centres, analysed the clinical data and the genotypes, and drafted the manuscript. FR coordinated the study in Luxembourg. ATV and CL performed the molecular biology assays and aligned the sequences. KF, FE, DP, CF, KVL, MLD and DV all contributed to the data and samples collections in their own HIV centre. JCS and PG participated in the design of the study and helped to draft the manuscript. All authors read and approved the final manuscript.

## Pre-publication history

The pre-publication history for this paper can be accessed here:


